# Low-Power, High Data Rate Transceiver System for Implantable Prostheses

**DOI:** 10.1155/2010/563903

**Published:** 2011-01-03

**Authors:** A. R. Kahn, E. Y. Chow, O. Abdel-Latief, P. P. Irazoqui

**Affiliations:** Weldon School of Biomedical Engineering, Purdue University, 206 S. Martin Jischke Drive, West Lafayette, IN 47907, USA

## Abstract

Wireless telemetry is crucial for long-term implantable neural recording systems. RF-encoded neurological signals often require high data-rates to transmit information from multiple electrodes with a sufficient sampling frequency and resolution. In this work, we quantify the effects of interferers and tissue attenuation on a wireless link for optimal design of future systems. The wireless link consists of an external receiver capable of demodulating FSK/OOK transmission at speeds up to 8 Mbps, with <1*e*-5 bit-error rate (BER) without error correction, and a fully implanted transmitter consuming about 1.05 mW. The external receiver is tested with the transmitter *in vivo* to show demodulation efficacy of the transcutaneous link at high data-rates. Transmitter/Receiver link BER is quantified in typical and controlled RF environments for ex vivo and *in vivo* performance.

## 1. Introduction

Increasing complexity in biomedical device development demands low-power, high data-rate, transcutaneous wireless-telemetry interfaces. The rapid development in brain-computer interfaces and novel treatment strategy for chronic neural disorders is rapidly improving patients' lives across the world. These developments have increased the data transmitted and processed by implanted devices. Glaucoma, quadriplegia, and epilepsy are just a few conditions that stand to benefit from a high data-rate wireless telemetry link to an implantable device. Glaucoma is the second leading cause of blindness and is estimated to affect 60.5 million people worldwide by 2010 [1]. A novel way to study this growing disease is by implanting an intraocular pressure-monitoring device in the eye [2]. Quadriplegia currently affects 200,000 people in the United States [3]. Brain-computer interfaces (BCI) monitor neural signals to allow quadriplegics control of computer equipment. Neuroprostheses with transcutaneous links are crucial due to a multitude of reasons including reduced risk of infection and increased patient mobility. Epilepsy, another widespread disease, affects approximately 1.6 million people in the United States [2, 4]. Approximately 37% of all epileptic patients are drug resistant and diagnosed with medically refractory epilepsy [5]. The advancement of technology allows the pursuit of novel implantable-device-based diagnosis, monitoring, and treatment methods. In many cases, these pursuits require a high data rate transcutaneous wireless data-link.

Various data transfer techniques have been explored, including radio frequency (RF) [6–10] and inductive [11] telemetry. Inductive telemetry operates via near-field coupling, which requires near-perfect alignment and only functions across short distances. RF telemetry operates through transmitting and receiving radiating fields which propagate over long distances and, in most cases are relatively orientation independent [9]. Furthermore, the bandwidth available in the RF frequency band allows for orders of magnitude higher data-rates when compared to inductive data transfer. Neural recording head-stages can benefit from an array of 16 electrodes, 10-bit resolution, and sampled at approximately 10 kHz. Currently, data bandwidth requirements limit the headstages to one channel. Therefore, the minimum raw data-rate for these devices is 1.6 Mbps; however, error correction/coding schemes, start/end/sync bits, and redundancy require additional bandwidth. One neural recording device presented in [6] operates at a data-rate of 157 kbps. This relatively low transmission rate is sufficient for this device as it only allows for one channel of wideband recording. The other channels are represented by threshold crossings in order to reduce bandwidth. In neurorecording applications, the implant has more stringent requirements than the external device, utilizing low power and less complex processing. The transceiver link is, therefore, optimized as an asymmetric link where the minimal power draw of the implanted transmitter is priority and the complexity is applied to the external receiver side. A custom-designed receiver architecture is used to decode the unlocked-carrier, noncoherent, FSK-modulated transmission from the implant. This paper demonstrates an asymmetrically designed custom transceiver, with an implanted transmitter, operating in the 2.4 GHz ISM band and achieving data rates of up to 8 Mbps with a bit-error-rate of 7.5*e*-5 at distances up to a meter. Performance is compared to prior work in a figure of merit (FOM) combining data-rate and bit-error rate (BER). Furthermore, the demonstrated telemetry system BER is analyzed in greater depth over comparable systems [6–10]. Free-space, interference, and anechoic performance is quantified for both bench-top and *in vivo* implementations in the goal of providing insight to future telemetry design.

## 2. Design and Methods

### 2.1. Transmitter

**Figure 1:**
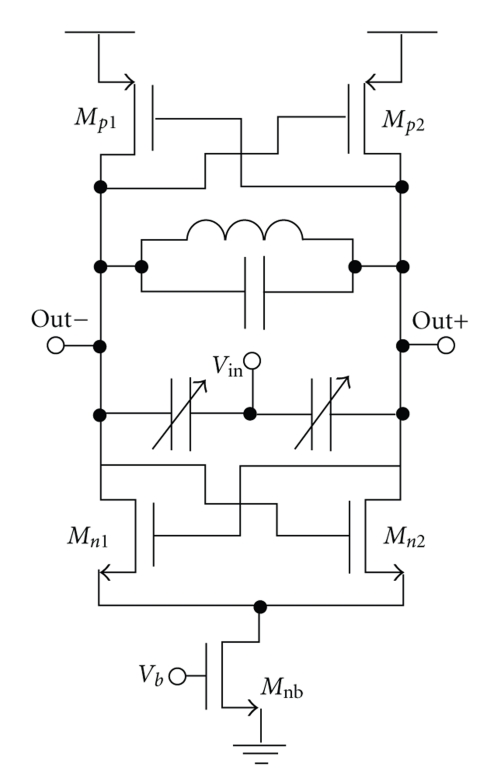
Complementary cross-coupled oscillator schematic.

Data is modulated onto a high-frequency 2.4 GHz carrier through a monolithic application-specific integrated circuit (ASIC) fabricated using the Texas Instruments 130 nm CMOS process. The ASIC transmitter consists of several blocks including the oscillator cross-coupled transistor pairs, varactors, inductor, and power amplifier. A voltage-controlled oscillator (VCO) is used to modulate the data through binary frequency-shift keying (BFSK). The complementary np cross-coupled VCO topology shown in Figure 1, is feasible for on-chip CMOS implementation, has quick startup times and is able to achieve low power consumptions while maintaining comparable or lower phase noise than other topologies [12]. In this oscillator type, the generated waveform stems from the resonance of an inductor-capacitor (*LC*) tank and uses the negative resistance principle to amplify and maintain the oscillation. The *LC* tank uses a inductor, *L* = 3.37 nH and a capacitor, *C* = 1.2 pF. Frequency modulation is performed through accumulation varactors placed in parallel with the *LC* tank. The *LC* tank capacitance variation with input voltage produces corresponding changes in the output frequency. 

On-chip inductor design on standard CMOS processes faces the challenges of capacitance coupling to the nearby substrate, size constraints, and energy loss via eddy currents generated on the substrate. Several design and layout methodologies are implemented to maximize the quality factor (*Q*) of the inductor, including an octagonal spiral, a patterned ground shield, and a differential (symmetric) topology [13]. A patterned ground shield, formed using strips of polysilicon, is included in the design to isolate the inductor from the substrate, reducing energy dissipation while minimizing image currents. The differential topology reduces the required area and the symmetry improves the *Q*, and thus overall phase noise, of the differential VCO. The final 200 *μ*m × 200 *μ*m inductor, designed and fabricated using the TI process, has a measured inductance of 3.37 nH and *Q* of 10.5.

The assembled VCO output is fed through a self-biased class AB power amplifier to buffer the oscillator from a potential varying load and to improve the output power of the transmitter. To optimize power transfer, an L-match network is used to conjugately match the transmitter output to the antenna.

**Figure 2:**
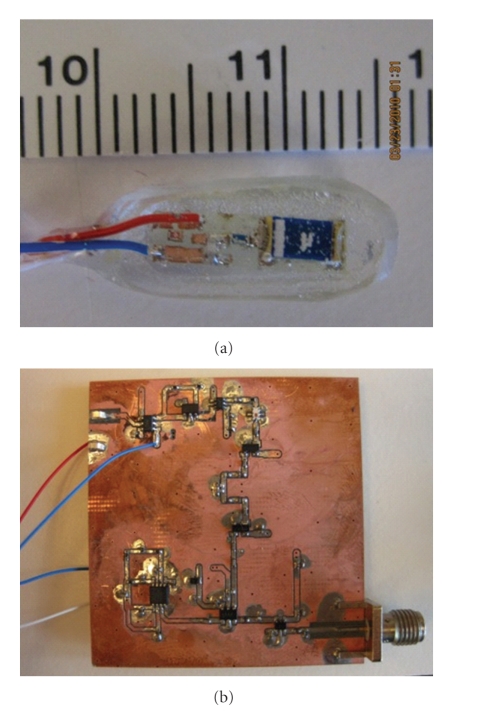
(a) Fabricated and encased transmitter system and (b) receiver Board.

A 6 mm × 16 mm PCB is designed to mount the ASIC 2.4 GHz transmitter, matching network, and Fractus Micro Reach 2.4 GHz chip antenna. The transmitter chip is wire bonded to the board and encased in a Hysol M-31CL clear medical grade epoxy as shown in Figure 2(a). BPFSK performance verification is assessed using an Agilent E4404B Spectrum Analyzer. When modulated at 8 Mbps, peaks are seen at 2.4 and 2.5 GHz with −45 dBm transmitted power at a distance of 30 cm.

### 2.2. External Receiver

**Figure 3:**
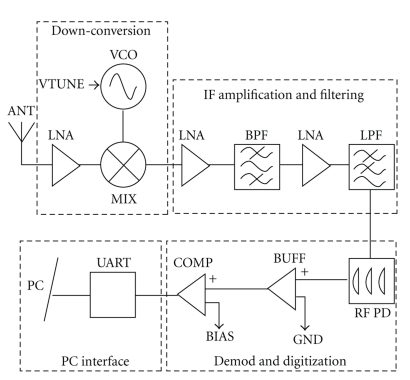
External receiver component schematic.

The external system, block diagram shown in Figure 3, demodulates the transmitted BFSK signal as a 2.5 GHz OOK signal, allowing greater flexibility as it can receive both FSK and OOK modulated signals. Analyzing half the signal, as done in OOK, produces an increase in rate of error, which is addressed later through filtering and error correction after acquisition. 

#### 2.2.1. Downconversion

Downconversion is desired for the significantly reduced power requirements in amplification and processing at lower frequencies. The down-conversion is accomplished through noncoherent, single-conversion, low-IF architecture [14]. The signal is fed through an L-matching network to a Maxim IC (MAX2644) low-noise amplifier (LNA). Radiation coupling on the high frequency interconnects is attenuated through the use of grounded coplanar waveguides (GCPW). Optimal matching design between components is verified, through modeling the GCPW characteristics by analytically determining the effective dielectric constant (*ε*_eff_) and characteristic impedance via an elliptical integration method presented in [15]. The resulting design is a matched waveguide with a width of 30 mil and space to ground plane depth of 10 mil. 

The 2.4/2.5 GHz signal is fed into the RF input port of a Maxim IC (MAX2680) double-balanced mixer and the local oscillator (LO) port is fed a −3 dBm, 2.38 GHz signal from a Maxim IC (MAX2750) VCO to create 20 MHz and 120 MHz intermediate frequencies (IF).

#### 2.2.2. IF Amplification and Filtering

After down-conversion, IF amplification and filtering/isolation is performed. The mixer IF output is first fed, through an L-match network, into a Maxim IC (MAX2650) LNA, providing 20 dB of gain. Optimal performance for our configuration is seen when the MAX2650 is operating at 3 volts, 1.5 volts below the recommended minimum VCC, likely due to noise figure reduction. 

The LNA output is then fed into a 120 MHz passive T-network 3-pole Butterworth band-pass filter. The 120 MHz centered, 20 MHz pass-band filter is designed through the insertion loss method described in [16]. The pass-band bandwidth is optimized to account for the transmitter frequency jitter resulting from the unlocked nature of the low-power VCO designed for this application, and the insertion loss in-band is −0.24 dB. An additional 20 dB of gain is provided using a second Maxim IC (MAX2650) LNA. Matching networks are designed, modeled, and implemented for each input/output from the amplifiers and filters. The result after amplification and filtering is an OOK-modulated, 120 MHz signal. Converting to an OOK signal allows the use of this receiver for both FSK and OOK modulation schemes at 2.4/2.5 GHz. The overall noise figure for the RF front-end and IF amplification is about 2 dB, determined by Friis' formula [14].

#### 2.2.3. RF Power Detection and Digitization

Demodulation of the OOK-modulated signal is performed through the use of a Linear Technology (LT5534) RF power detector. The monolithic power detector is configured to operate on a 3 V source and detect signals down to −60 dBm over a frequency range of 50 MHz to 3 GHz. Input impedance is matched at 120 MHz, providing an S21 of −4.25 dB. Logic high occurs during 120 MHz, and logic low occurs when low RF energy is present in the detection band. The 500 MHz passive low-pass filter is used prior to the power detector to decrease the high frequency out-of-band noise. An Analog Devices (AD8061) high-speed buffer placed after the power detector amplifies the signal and drives the high input impedance of the comparator. The buffer circuitry is modeled using ADISim by Analog Devices. 

A Linear Technology (LT1719), high-speed comparator performs digitization of the signal. The comparator bias is set to 2 V and generates a 3 V logic high (digital 1) when 120 MHz is present and 0 V (digital 0) otherwise. The high-speed buffer and comparator are critical to achieve and maintain the integrity of a digital signal. 

#### 2.2.4. Receiver Construction

The external receiver is comprised of discrete components integrated on a PCB, as shown in Figure 2(b). The PCB is designed in both Allegro and Camtastic 99!, and constructed though an Accurate 360 CNC milling machine. Vias, 0.28 mm in diameter, are electroplated to achieve a wall thickness of about 400 *μ*m. After electroplating, copper insulation and rubout is completed. Insulation is performed first on the top layer with sequentially increasing routing bit diameters to provide a deep space with clean edges, which minimizes the occurrence of electrical shorts. 

Individual components are attached in sequence following the signal path while the RF characteristics are quantified stage-by-stage before subsequent components are attached. An RF probe, first calibrated down to the measurement point, is used to determine the gain/attenuation at the output of each component after its integration onto the board. Each stage is matched to a standard 50 ohm impedance, allowing the 50 ohm RF probe to mimic the output load of subsequent components.

### 2.3. Data Acquisition and Processing

The serial stream of digital data is sent to a Universal Asynchronous Receiver/Transmitter (UART), converting from digital to USB, which enables interfacing with a PC. An FTDI USB module (FT4232H) is used to read the serial digital data-stream into the computer. The USB chip is configured to use the serial peripheral interface (SPI) bus protocol to read data at a sample rate of 10, 15, and 30 MSps for 1 Mbps, 2/4 Mbps, and 6/8 Mbps signals. Data streamed through the SPI is digitally filtered and processed on a PC.

Data acquisition is performed through a VC#.NET program using FDTI drivers. The total number of windows is defined as 100*e*6 in the UART; however, it is decreased due to a clock delay during every 8th sample in the UART hardware. Data window length is determined by the maximum limit of the UART before a 5 ms delay occurs. The delay is accounted for by acquiring an ideal signal through the USB interface for each data-rate. The ideal data is processed by the filtering program to determine the total number of expected bits at each data-rate at a given sampling frequency and measurement window. Acquisition processing is performed through a four-point digital moving average filter to eliminate spurious noise bits. Once filtered, the data is re-digitized with a 0.5 high/low threshold.

### 2.4. Bit-Error Rate Testing

The BER is a measure of receiver efficacy without considering the effects of the system link. The quantity is determined by calculating the number of transmission errors over the total number of bits transmitted [17] as follows:

(1) $$\text{BER}=\frac{\sum \text{error}\;\text{bits}}{\sum \text{transmitted}\;\text{bits}}.$$

The resulting ratio (1) is determined by summing the error bits that occur within in the received transmitted bit population. The method used to calculate asynchronous BER in the constructed receiver takes into account error that is introduced by nonideal sampling of the UART

(2) $$\frac{\sum \text{transmitted}\;\text{bits}}{153\;\text{windows}}=\frac{\text{Average}\;\text{bits}}{\text{window}}.$$

An Agilent MXG Signal Generator, operating at the desired data-rate, serves as a control transmit signal. The quantification of transmitted bits is calculated for each data-rate by sampling >8 million bits over 153 data windows. All windows are equal in length and the average number of bits in each window is calculated (2). The resulting average is used as the denominator in (1) in order to calculate the BER of the received signal. The difference between the number of obtained bits and the number of expected bits is the number of errors acquired during receiving.

### 2.5. Testing and *In Vivo* Experimentation

**Figure 4:**
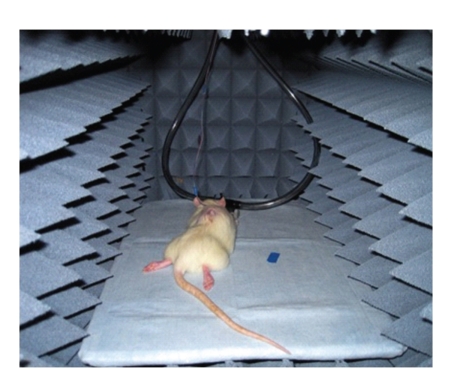
*In Vivo* anechoic testing chamber.

Anechoic testing is performed in a rectangular 45.72 × 45.72 × 152.4 cm anechoic chamber. The chamber is composed of a plywood support shell, a copper sheet isolation shell, and a pyramidal foam attenuating shell (Figure 4). The Cuming Microwave C-RAM SFC-4 foam attenuating shell operates optimally at frequency ranges greater than 1 GHz [18].

The wireless link between the external receiver and implantable transmitter is tested in open-air, an anechoic chamber, and in an anechoic chamber with a Bluetooth interferer present. Antenna orientation is maintained throughout the tests, the implantable transmitter lying flat, with the antenna directed toward the receiver. The receiver is elevated approximately 15 cm to maximize the Fractus antenna propagation pattern. 

Testing is performed with the receiver in one half of the chamber and the transmitter in the other half of the chamber. Bluetooth interference is generated through a cell-phone and Bluetooth headset streaming audio, and placed at the junction point of the two halves of the chamber. BER is determined in each experimental setup at 50 cm and 100 cm for 1 Mbps, 2 Mbps, 4 Mbps, 6 Mbps, and 8 Mbps. A total of three trials are completed to generate an average and standard deviation. 

**Figure 5:**
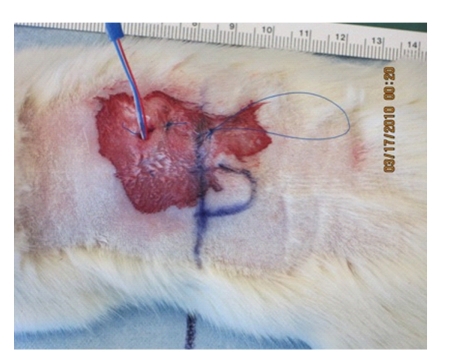
Transceiver implantation site (the vertical line represents T-10, half oval is a marked outline of the device on the skin).

The Purdue Animal Care and Use Committee (PACUC) approved all animal procedures. The procedures adhere to the NIH Guide for the Care and Use of Laboratory Animals. *In vivo* testing is performed following transcutaneous implantation of the transmitter in a rat model. The subject is given isoflurane as an anesthetic and 0.05 mg/kg butorphanol as an analgesic. Isoflurane is continually administered throughout the experimental procedure to maintain an anesthetic plane. The transceiver is placed under the dorsal skin, 8.5 mm lateral from the T-10 vertebrate (Figure 5). 

The implant region skin thickness was approximately 1 mm throughout all trials. The rat dorsal skinimplant location was chosen for repeatability in future trials. Rat dorsal skin is, on average, thicker than the human scalp [19]. Therefore, the rat dorsal skin serves as a the worst-case implant scenario in which the transmitter/receiver combination performance limits can be quantified. The transmitter frequency was verified with an Agilent Spectrum Analyzer prior to implantation. Postoperatively, the wound was flushed with saline and sutured closed with Ethicon Prolene polypropylene suture. During the open-air experiments, the antenna is positioned laterally on a plastic mount at 50 and 100 cm from the subject. Both isolated and Bluetooth interferer experiments are performed with the subject 50 cm inside one half of an anechoic chamber, and the receiver located in the other half. Testing is performed with both chambers closed together and wire mesh placed around the seal to reduce leakage. 

During the anechoic chamber experiments, local Wi-Fi signal strength and local Bluetooth sources are reduced to minimize power leakage into the test chamber.

## 3. Results

**Figure 6:**
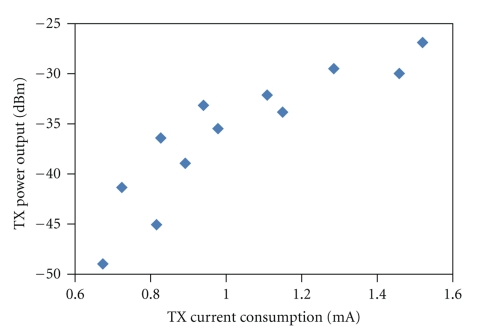
Measured transmitter output power as a function of current consumption.

The transceiver specifications are summarized in Table 1. The asymmetric transceiver design methodology is clearly seen in the power consumption measurements. The transmitter output power as a function of current consumption is plotted in Figure 6. When the implantable transmitter is set to its lowest stable output power setting of −41 dBm, the measured current consumption is 700 *μ*A from a 1.5 V supply. The external receiver is able to successfully demodulate the data from this low-power transmitter while consuming 150.6 mW, which is acceptable due to the larger power supply options on the external side. 

The receiver sensitivity, BER, and maximum data-rate performance are all correlated and inversely proportional to one another. Therefore, optimization of the parameters is performed to determine a point with maximum data-rate while maintaining a high sensitivity. An optimal point for our target application is found at a data-rate of 8 Mbps, a BER of 7.5*e*-5, and a sensitivity of −80 dBm. To achieve statistically relevant results, over 1e6 bits are tested to accurately quantify the receiver BER. 

**Figure 7:**
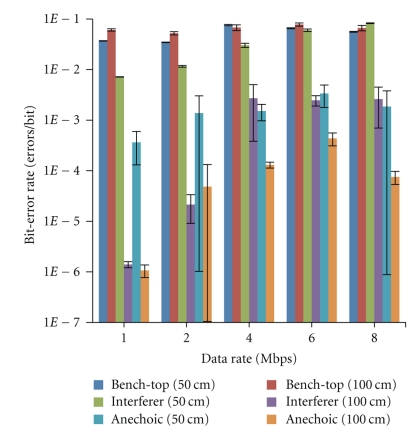
Free-space bit-error rate for lab bench-top, Bluetooth interferer, and anechoic chamber testing at 50 cm and 100 cm. The error bars at each data-point represents the standard deviation over three measured trials and the points are offset slightly so that error bars are viewable.

The free-space testing results are plotted in Figure 7 and represent the following cases: in open-air on the lab bench-top, in the anechoic chamber, and in the anechoic chamber with an active Bluetooth interferer. In each case, measurements are taken at transmitter-receiver separation distances of 50 cm and 100 cm and data-rate is swept from 1 Mbps to 8 Mbps. Three measurements are taken for each parameter set, and the results are averaged and plotted in Figure 7 along with the corresponding standard deviations shown as error bars. At 8 Mbps, the lowest BER case is for the measurements taken in the anechoic chamber for the separation distance of 100 cm, which have an average value of 7.5*e*-5 errors/bit. The next lowest BER case is the measurement taken in the anechoic chamber at 50 cm followed closely by that in the chamber at 100 cm but with an interferer. The next two BER cases are the ones taken on the lab bench-top, and the worst case for errors at 8 Mbps is that measured in the anechoic chamber with an interferer at a distance of 50 cm.

**Figure 8:**
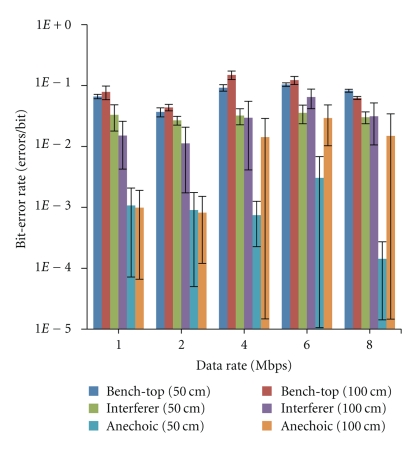
*In vivo* bit-error rate for bench-top, Bluetooth interferer and anechoic chamber testing at 50 cm and 100 cm. The error bars at each data-point represents the standard deviation over the three *in vivo* trials and the points are offset slightly so that error bars are viewable.

Results from the *in vivo* studies are plotted in Figure 8 where the BER is plotted for various data-rates at the same 50 cm and 100 cm transmitter-receiver separations and data-rate range of 1 Mbps to 8 Mbps. As in the case of free-space testing, the BER is measured in the *in vivo* studies for the following cases: lab bench-top, anechoic chamber, and chamber with Bluetooth interferer tests. For each case, 3 *in vivo* animal trials are performed to achieve the averages plotted in Figure 8 and the standard deviations represented in the error bars.

## 4. Discussion

### 4.1. Transmitter Power Consumption

For testing reliability, the transmitter is programmed to output a measured −14 dBm to the antenna. It is important to determine how the measured BER data relates to the lower power case. To maintain a wireless data-link and account for a reduction in output power, either the BER tolerances must increase, antenna efficiency must improve, or transmit distance decreased [17]. Friis transmission formula is used to determine the reduction in wireless-link distance accounting for a decrease in transmitter output power

(3) $$P_{\text{RX}}=\frac{G_{\text{TX}}G_{\text{RX}}P_{\text{TX}}\lambda^{2}}{{\left(4\pi D\right)}^{2}},$$

where the subscripts RX and TX denote parameters of the receiver and transmitter, respectively, *G* is the gain of the antennas, and *D* is the distance between the transmit and receive antennas.

For a programmed current consumption of 700 *μ*A, the measured output power is −41 dBm, which will result in a decreased distance to maintain performance. To maintain the same operating distance, change in BER as a function of distance, is evaluated in Figures 7 and 8. In the case of lab bench-top testing in free space at 8 Mbps and 700 *μ*A implanted transmitter current consumption, the BER is calculated to increase by a factor of 2.665 when increasing distance from 50 cm to 100 cm.

### 4.2. Receiver

The receiver performance characteristics are compared to other wireless, neuroprosthetic receiver-links. Maximizing data-rate and minimizing consumed implantable transceiver power are both important factors when considering wireless RF-links. It is with these two goals in mind that lead to the determination of a figure of merit (FOM), where

(4) $$\text{FOM}=\frac{\text{Data}\;\text{Rate}\;\left(\text{Mbps}\right)}{\text{Consumed}\;\text{Power}\;\left(\text{mW}\right)}.$$

Comparing the derived FOM against other devices shows the proposed system has superior performance when considering data-rate and power. A comparable implantable device operating at a 6 Mbps interface has a transmitter that consumes 2 order-of-magnitude more power than the system outlined in this report. As seen in Table 2, this relatively high power consumption is reflected in a low FOM.

### 4.3. Free-Space Testing

An anechoic chamber is important for consistent and reliable transceiver testing; however, lab bench-top testing also provides useful insight as it simulates a clinical setting where multiple interference sources may be present. The results from the free-space testing, plotted in Figure 7, show a reduction in BER of more than 2 orders of magnitude when comparing the bench-top to the anechoic chamber measurements. The reduction is due to Wi-Fi, Bluetooth, and other RF interference sources apparent in the bench-top tests while attenuated in the anechoic chamber tests.

Simulating a single but close proximity interference source, a Bluetooth transmitter is placed at the center point between the transmitter and receiver. In this test, there is a higher BER when the transmitter and receiver are closer together, this is primarily due to the decreased distance between the interferer and the receiver. From the measured data, plotted in Figure 7, with the Bluetooth interferer at the center, there is at least an order-of-magnitude greater BER when the transmitter and receiver are 50 cm apart versus when they are 100 cm apart.

It is interesting to note that the BER measurements in the anechoic chamber do not yield an increase in BER when the transmitter-receiver separation distance is increased even though received signal strength (RSS) is reduced. This effect is likely due to close proximity of the devices producing reflective surfaces, which increase multipath interference. When the transmitter and receiver are further apart, the multipath effects are greatly reduced contributing to a reduced BER. In the anechoic chamber measured results, there is an orders of magnitude reduction in BER when separation distance is increased from 50 cm to 100 cm.

Increasing BER with increasing data-rates is relatively consistent through all trials. Various factors may contribute to this effect stemming from the transmitter, receiver, and the data acquisition/processing. On the transmitter side, there are upconverted noise effects, frequency shifting delays, and a wider primary sinc function lobe when examining the frequency domain of a higher data-rate square wave convoluted with a 2.4 GHz carrier. The receiver side will face reduction in signal-to-noise characteristics of the transmitted signal. With increasing data-rate, the data acquisition will also have an increasingly difficult time discerning a digital 1 from a 0 due to inherent limits of sampling rate.

### 4.4. *In Vivo*

For a given distance, the BER from *in vivo* testing is higher than the BER from ex vivo testing due to the reduced signal strength from tissue-induced power loss [20]. Similar to the free-space testing results, the BER at 50 cm and 100 cm in the anechoic, chamber is significantly less than the lab bench-top and the Bluetooth interferer results. Due to the reduced SNR during the *in vivo* test, the interferer has a greater effect at both 100 cm and 50 cm. There is an uncharacteristic reduction in BER during the *in vivo *8 Mbps, 100 cm, anechoic and Bluetooth interferer cases, which results from the frequency hopping nature of Bluetooth interferers. At higher data-rates, the size of the window sample is smaller, decreasing the probability of a hopping Bluetooth carrier frequency overlapping the signal. This effect is not limited to our particular test procedure and is a potential method for achieving lower BER for high data-rate transceiver links.

An additional factor contributing to a sharp change in BER when increasing from 2 Mbps to 4 Mbps in all test setups is UART sampling rate. Changing a memory register in the VC# program sets the UART sample rate; however, it is limited to integer divisions of 30 MHz. It is because of this that both 2 Mbps and 4 Mbps data-streams are acquired at 15 MSps. Half the number of bit defining samples for 4 Mbps contributes to the sharp increase in error. Less samples yield a more noise susceptible data-stream. 

Currently, implantable medical devices primarily utilize the 402–405 MHz Medical Implant Communications Service (MICS) band for wireless telemetry. Validating the utility of our transceiver in implantable medical devices, the same BER test case parameters are used in the Zarlink MICS-band device testing except for the data-rate due to the fact that the Zarlink device only achieves a maximum raw data-rate of 500 kbps. This commercially available component does not utilize an asymmetric design, and, thus, the net consumption of the device is 5 mA, which yields a FOM of 0.16 Mbps/mW. During the Zarlink test, BER measurements in the anechoic chamber are actually slightly worse than those on the bench-top. This result is due to the fact that the 400 MHz band is not widely used and therefore the anechoic chamber offers little interference shielding benefit and introduces reflection-based multipathing, which increases the rate of errors. The Zarlink free-space BER measured on the bench-top and within the anechoic chamber is on the order of 1*e*-5, which is higher than that of our device in free space, in the anechoic chamber, at 1 Mbps and 100 cm. The *in vivo *Zarlink BER is only slightly higher than the free-space testing and is on approximately the same order of 1*e*-5. This BER is less than the *in viv*o results of our 2.4 GHz device and is attributed to the lower tissue-induced signal loss at 400 MHz. When examining only BER in an *in vivo* setting, the MICS band offers more advantages due to reduced tissue-induced power loss and fewer interferers. However, when considering bandwidth, power output, and antenna size, the 2.4 GHz band is superior over MICS. The unlicensed 2.4 GHz band also results in less strict protocols, procedures, and requirements.

## 5. Conclusion

The effect of interferers and tissue-attenuation on a wireless receiver suited for high data-rate, low-power communication with implantable biomedical devices is quantified. The external device is shown to be capable of demodulating 8 Mbps with the RF-transmitter link. A high data-rate is beneficial in error-correction schemes to be employed to reduce BER. Characterization of the designed receiver yields a better than 1*e*-5 BER at the maximum data-transmission of 8 Mbps, providing an excellent method for both burst transmission applications and high data-rate neural-prostheses transmission. The proposed external receiver system achieves a higher data-rate per unit power over comparable cutting edge transmission systems. Quantification of performance for the device shows interference in the communication band to have a greater effect over attenuation due to distance. Analysis of MICS yields a trade-off to determine data bandwidth requirements and implantable device size. Optimal performance may be achieved at a higher frequency outside of the heavily used 2.4 GHz band to benefit from maximum data transfer, lack of interferers and antenna size.

## Figures and Tables

**Table 1:** Transceiver specifications.

Specification	Measured Value
Operating frequency	2.4/2.5 GHz
TX power consumption	1.05 mW
TX output power	−41 dBm
TX phase noise	81.8 dBc/Hz @ 100 KHz
	−103.28 dBc/Hz @ 1 MHz
TX inductor *Q *	10.5
RX power consumption	150.6 mW
Modes of reception	BSFK/OOK
RX selectivity^a^	−3 dB @ 20 MHz
RX sensitivity	<−80 dBm
Data-rate	8 Mbps
Communication distance	>1 m
BER @ 8 Mbps, −80 dBm^b^	7.5*e*-5 Errors/bit

^a^The selectivity bandwidth of the receiver is intentionally designed to be wide 20 MHz to account for the unlocked oscillator transmitter jitter.

^b^Over 1e6 bits are tested for the error-rate quantification.

**Table 2:** Figure of merit for tranceiver systems.

Paper	Parameters			
	Data-rate (Mbps)	Transmitter power consumption (mW)	Frequency (MHz)	FOM
This work	8	1.05	2400/2500	7.619
R. Harrison [6]	0.157	0.5	902–928	0.314
J.A.B. Gerald [8]	0.1	50	1/1.067	0.002
